# Urban riverbank green spaces as therapeutic environments: Examining the psychosocial benefits of square dance in aging populations in China

**DOI:** 10.1016/j.ssmph.2025.101848

**Published:** 2025-07-24

**Authors:** Song Wei, Hongli Yu, Chen Sun

**Affiliations:** a College of Physical Education and Health Science, Yibin University, Yibin, 644000, Sichuan, China; b College of Physical Education, Sichuan University of Science & Engineering, Zigong, 643000, Sichuan, China

**Keywords:** Psychological health, Anxiety, Depression, Stress, Life quality, Older adults

## Abstract

Green spaces along urban rivers have become popular venues for promoting physical activity (PA), particularly among older adults. However, there is little research on how the timing (morning versus nighttime) and location (green versus non-green spaces) of square dancing (reflecting PA) influence psychosocial benefits for older adults. Furthermore, gender differences in these interactions remain unexplored. This study examines the psychosocial benefits (e.g., reducing anxiety and depression disorders, improving psychological well-being and quality of life, enhancing emotional well-being, and alleviating psychological stress) of square dancing for aging Chinese adults with gender-specific preferences. This study utilized a cross-sectional sampling method with older adults from urban riverbank green spaces in China (N = 536; 315 women and 221 men) in 2023. We collected demographics, preferences (both time and location), and psychosocial health outcomes using well-known tools. Statistical analyses included correlation assessments, Wald Chi-Square tests, and gender-specific comparisons. Results indicated that 71.08 % of participants preferred PA in green spaces and 60.82 % preferred nighttime activities, highlighting clear patterns of engagement. Among men, PA timing was more strongly related to anxiety levels (Wald χ^2^ = 5.178). Conversely, PA location was significantly associated with emotional well-being (Wald χ^2^ = 5.822) and quality of life (Wald χ^2^ = 6.748) outcomes among women. Nighttime PA in urban riverside green spaces offers psychological benefits to older Chinese adults, highlighting distinct gender- and time-specific effects. Considering these findings, public health and community planning initiatives need to be customized to improve overall health and well-being among the aging population.

## Highlights

• Nighttime square dancing reduces anxiety levels among older men.
• Green-space activity boosts emotional well-being among older women.
• Gender motivations shape square dancing participation.
• Social bonds mediates the benefits of green-space group activities.
• The design of safe, inclusive green spaces aids psychosocial health.

## 1 Introduction

The aging population has significant impacts on demographic transformation worldwide (Abdul Jabbar et al., 2025), affecting public health systems, public policy, and community planning initiatives (Ko et al., 2025; Lu et al., 2025). Among the fastest-growing elderly countries in the world, China faces significant challenges in preserving its health and enhancing the quality of life for its growing elderly population. The National Bureau of Statistics of China reported that individuals aged 60 and over comprised approximately 18.7 % of the population in 2020 (National Bureau of Statistics, 2021). The projections indicate that this figure may exceed 30 % by 2050. Considering this demographic shift, it is imperative that effective, accessible, and culturally relevant solutions be developed to promote healthy aging. Elderly people suffer from chronic illnesses and psychological challenges (Menotti & Puddu, 2025). Physical activity (PA) is widely recognized for its positive effects on physical and mental health throughout life, and it has been specifically proven to benefit aging people through extensive research (Shi et al., 2024). PA is consistently associated with reduced risks of cardiovascular disease, functional decline, obesity, diabetes, and certain cancers (Neil-Sztramko et al., 2022). Additionally, it promotes mental health and cognitive function in older adults (VaezMousavi et al., 2025). Furthermore, numerous clinical and epidemiological studies have indicated that PA can significantly alleviate depression, anxiety, and stress (Atchison et al., 2024; Kim et al., 2024; Yılmaz Koğar & Koğar, 2024). Aside from this, PA improves mental health and quality of life.

Recent studies have revealed that public administration, particularly access to green spaces, has significant impacts on health outcomes (Kong et al., 2025). It has been demonstrated that green spaces, such as gardens, parks, and riverbanks, provide additional emotional, psychological, and social health benefits that extend beyond those of PA alone (Ai et al., 2025; Deng et al., 2025; Yuan & Chen, 2025). It is argued that natural environments can reduce physiological stress responses, alleviate mental fatigue (Zhang et al., 2024), and promote emotional recovery in accordance with theories such as Attention Restoration Theory and Stress Reduction Theory (Elkins et al., 2021). Recently, urban riverside green spaces have become recognized as ideal settings for PA because they are visually appealing, easily accessible, and foster community connections among older adults (Serra & Feio, 2024). Throughout China, activities such as walking, Tai Chi, and culturally specific group exercises, such as square dancing, have become increasingly popular. As a result of these activities, aging individuals can maintain their physical well-being and ability to engage in social activities. Particularly, square dancing combines physical exercise, music, and social interaction, making it particularly beneficial for older adults living in cities.

Green spaces promote health for older individuals (Petersen et al., 2018; Wu et al., 2024). However, significant gaps remain in understanding the impact of PA timing in these environments on mental and physical health outcomes for square dancing. Studies to date have focused on the overall benefits of PA in conjunction with nature-based features (Cook et al., 2025). However, few studies have examined the temporal factors associated with variations in psychosocial and physiological responses among older adults who participate in morning versus evening activities. Nighttime physical activities are increasingly common in urban China, influenced by sociocultural factors such as family caregiving responsibilities and social customs (Wu et al., 2023; Zhang et al., 2022). While nighttime activities present unique challenges regarding safety, visibility, and perceived risk, some older individuals may find sustained PA difficult or impossible. Furthermore, research specifically comparing nighttime PA benefits to morning activities in this context is lacking. Further study of potential benefits such as reduced anxiety and depression, improved emotional well-being, and enhanced overall quality of life is necessary (Villarreal-Zegarra et al., 2025). Furthermore, differences between male and female preferences for PA timing and location, as well as their effects on health outcomes, have not been thoroughly investigated in square dancing. Considering differences in societal roles, obligations, and safety concerns, men and women may perceive and interact differently in green spaces. Developing inclusive approaches that cater to the diverse needs of older adults requires an understanding of these gender-specific insights.

Comparatively, global studies have sometimes linked environmental factors, such as greenery and safety (Cook et al., 2025; Serra & Feio, 2024), to exercise compliance without considering culturally specific activities, such as square dancing. Global literature and Chinese literature differ significantly in emphasis and methodology (Ionescu et al., 2025; Ye et al., 2025). Western research focuses on quantitative assessments of stress and mood, often neglecting community-based activities (Friedrich et al., 2024; Harris et al., 2023; Yılmaz Koğar & Koğar, 2024). Chinese studies emphasize social benefits without a comprehensive environmental analysis (Yuan et al., 2023; Zhao et al., 2024). Worldwide, people overlook riverbank settings as therapeutic venues, and China research lacks cultural psychology perspectives. Furthermore, multidisciplinary collaboration has significant gaps; for instance, aging research rarely interacts with planning research. Our understanding of the long-term effects of these interactions is limited due to the lack of longitudinal and mixed methods approaches, as well as other methodological limitations.

The demographic aging and rapid urbanization of the Chinese population make it imperative to understand how elderly health outcomes are affected by their involvement in urban riverside green areas (Song et al., 2025), particularly in terms of PA timing and location. Those studies can provide insight into health promotion practices and environmental modifications to better serve older adults (Wang & Boros, 2025). It will be valuable to healthcare practitioners, community planners, and policymakers. In investigating the often-overlooked variable of exercise timing—morning or night—the research contributes significantly to our understanding of the physical and mental health effects of PA. Providing culturally relevant and effective recommendations, the study can examine various cultural practices, such as square dancing, within the distinct context of urban China. In addition, such studies can provide detailed insight into gender disparities (Lu et al., 2025). Such data can be used to inform inclusive community initiatives and urban design guidelines (Li et al., 2025). Ultimately, such research may improve health outcomes among diverse older adult populations by encouraging broader participation.

**Fig. 1:**
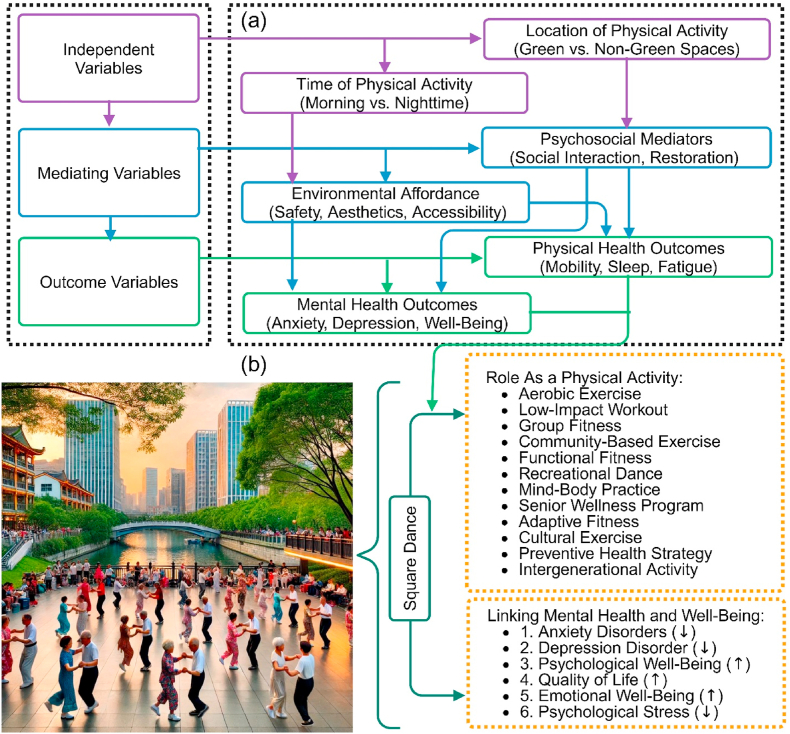
Panel (a) presents the hypothesized model, while panel (b) provides an image illustrating square dancing and its effects on physical activity, psychological health, and overall well-being among older adults.

Specifically, this research explored the intersection of three underexplored topics: riparian green spaces, square dancing, and aging demographics. We applied Western therapeutic approaches in a Chinese setting, highlighting collective cultural values (Fig. 1). It explores how riverside design enhances the psychological benefits of square dancing by focusing on gender differences. This addresses gaps in both global and Chinese literature. Furthermore, it provides policy recommendations for the creation of age-friendly urban green spaces that align with goals for health equity and urbanization in China. It is necessary to conduct research to evaluate the effectiveness of urban riverbank green spaces compared with other types of non-green spaces. This research aims to (1) examine the psychosocial benefits of square dance in aging populations within urban riverfront green spaces in China; (2) it aims to identify improved psychological health, enhanced well-being, and increased resilience that influence the relationship between PA and health outcomes; and (3) the study assesses gender- and location-specific differences in PA timing preferences and their corresponding health impacts. This study examines how timing (morning vs. night) and setting (green vs. non-green spaces) affect older adults’ psychosocial health, with attention to gender differences. It hypothesizes that the temporal and spatial contexts of square-dancing shape well-being through gender-specific pathways.

## 2 Materials and methods

### 2.1 Study area

Mianyang is the second-largest city in Sichuan Province in China, located at 31°28′N and 104°41′E (Jian & Hao, 2020). In western China, it stretches 20,249 km^2^ along the Fujiang River, a significant tributary of the Yangtze River. In Mianyang, 22.3 % of residents aged 60 or older, which was higher than the national average of 18.7 % (National Bureau of Statistics, 2021). Studies on aging and health are particularly relevant here. Its urbanization index stands at 72.1 %. However, 41.3 % of green space is present in this urban area. There are 140 km of open spaces along the Fujiang River, which serves as a natural axis for the urban center (Sichuan Provincial Bureau of Statistics, 2021). They include parks and corridors designed to enhance recreational enjoyment, promote public health, and ensure ecological sustainability (Cao et al., 2021). The landscape of this city promotes PA among older adults through key environmental and social factors (Zhou, 2024). The first attribute is biophilic design, which integrates natural visuals and sounds. The sight of flowering *Nelumbo nucifera* and the sound of flowing water can inspire mental rejuvenation. The elderly benefit from water proximity by reducing perceived stress. This design emphasizes safety and accessibility. The nighttime lighting conditions are highly rated by users. Visitor security measures, like emergency call boxes and surveillance cameras, address common concerns. Additionally, open lawns and amphitheater-style seating promote community interaction. Many of these spaces host Tai Chi clubs, fan-dancing clubs, and informal social walks, all of which encourage intergenerational engagement. Cultural practices and environmental influences affect how these riverfront sites are used throughout the day. Most older adults who exercise between 5:30 and 8:30 a.m. do Qigong, brisk walking, or square dancing. Temperatures around 18 °C in the summer align with the body's natural cortisol cycle, enhancing metabolic efficiency. Leisure walks and social interactions through square dancing are popular among older individuals between 7:00 and 9:30 p.m. Nighttime activities benefit from reduced noise levels and increased humidity, further increasing sensory relaxation. As part of its age-friendly initiatives, the city sponsors exercise classes for older adults under the "Silver Hair Fitness Initiative".

### 2.2 Research design and subjects

Researchers used a cross-sectional approach to study community dwellings for older adults residing in an urban environment with riverside green spaces in Mianyang. Researchers collected data on square dancing activities (reflecting PA) from September 1 to November 30, 2023. Participants were recruited through a combination of community outreach strategies, including collaboration with local professional and random square dance groups. Among the criteria for eligibility, participants were required to be at least 60 years old, have lived in the city for at least one year prior to their study, possess the ability to complete self-reported questionnaires independently with minimal assistance, and engage in outdoor PA through square dance at least once a week in designated urban riverbank green spaces. Exclusion criteria included confirmed neurodegenerative conditions such as Parkinson's disease and Alzheimer's disease. Additionally, any medical contraindications to exercise, such as significant cardiovascular instability, and incomplete or inconsistent responses to survey questions were also excluded. This study enrolled 536 older adults pursuant to the written consent process and ethical guidelines of the 2013 Declaration of Helsinki. No minors were involved in this study. The institutional review board of the corresponding university approved the project on November 17, 2022. Among these participants, 210 (39.18 %) preferred square dancing in the morning, whereas 381 (71.08 %) preferred activities at night. In general, most respondents reported managing at least one chronic health condition, such as hypertension or diabetes. It is consistent with national prevalence estimates for older Chinese adults (National Bureau of Statistics, 2021). The study was designed to ensure representativeness of the broader urban elderly population. Sample quotas were used to assess variations in psychosocial conditions and access to green spaces within neighborhoods. Instead of cash compensation, the participants received feedback on their PA and overall well-being.

### 2.3 Measures

The current study used several validated instruments (Ainsworth et al., 2015) to examine the relationship between the time and location of square dance (PA) and health outcomes among older adults. All methods were selected because they were reliable, relevant to ageing populations, and had previously been validated in Chinese community settings (Jiang et al., 2025; Li et al., 2022). Under study variables, these tools assessed various factors, including PA behaviors, mental health status, and physical function. The frequency, duration, and intensity of PA were assessed using a modified, structured version of the PA Scale for the Elderly (PASE), a widely recognized tool for evaluating PA in older adults (Hatami et al., 2021; Sia et al., 2023; Wiśniowska-Szurlej et al., 2020). The questionnaire included extra sections in addition to the standard PASE items. These sections were designed to capture the typical time during which PA occurs—classified as morning (6:00 to 8:00 a.m.) or nighttime (7:00 to 9:00 PM)—as well as the primary setting, focusing on urban riverbank green spaces compared with non-green spaces. Participants reported the types of activities they participated in (e.g., square dancing), the frequency of participation (days per week), and the average duration of each activity. Our composite PA scores were created using the PASE scoring algorithm. This helped us to have a deeper understanding of habitual behavior in relation to time and the environment.

The mental health of the participants was assessed using three validated instruments (Costantini et al., 2021; Fonseca-Pedrero et al., 2023; Ford et al., 2020). To assess the severity of depression disorders, the researchers used Patient Health Questionnaire-9 (PHQ-9). The PHQ-9 is a concise, binary-response questionnaire with a 4-point Likert scale specifically designed for older populations (Kim et al., 2024; Korsen; gerrish, 2022; Stanyte et al., 2023), where 1 represents never and 4 represents almost always. This measure assessed depression symptoms in the past two weeks. Some of these symptoms include anhedonia, low mood, sleep disturbances, and appetite fluctuations. Chinese versions of the PHQ-9 have demonstrated strong internal consistency (Cronbach's α = 0.87) and robust construct validity across samples. The cut-off score for identifying probable depression disorder was 4. Symptoms of anxiety disorders were assessed using the Generalized Anxiety Disorder-7 (GAD-7) scale (Atchison et al., 2024; Gottschling et al., 2020; Picconi et al., 2023), which consists of 7 items rated on a Likert scale from 1 ("not at all") to 4 ("almost every day"). Chinese adaptations of the GAD-7 have been validated in older populations and are regarded as reliable indicators of generalized anxiety disorders (Delamain et al., 2024; Do et al., 2022). Mental well-being was assessed using the World Health Organization-Five Well-Being Index (WHO-5) (Carrozzino et al., 2022; Long et al., 2021; Sischka et al., 2025), a brief instrument that includes 5 positively worded items rated on a 6-point scale (1 = "never" to 6 = "always"). The total score reflects psychological and emotional well-being. The Chinese version of the WHO-5 has excellent psychometric properties, with Cronbach's alpha values exceeding 0.90.

Physical health status was assessed both subjectively and objectively. The self-rated health of participants was evaluated using a single-item measure from the Medical Outcomes Study Short Form-36 (SF-36) (Açma et al., 2022; Grönkvist et al., 2024; Posada de la Paz et al., 2022), which asked participants to rate their overall health as excellent, very good, good, fair, or poor. In older populations, this measurement has consistently shown predictive validity for morbidity and mortality. Additionally, participants were asked to report the presence or absence of common age-related conditions such as diabetes, high blood pressure, arthritis, or cardiovascular disease on a chronic disease checklist.

Our research team collected information about sociodemographic and contextual factors using a general information form. As part of this form, variables such as gender distribution, age, education, living conditions, employment conditions, body function pain, and psychosocial conditions were collected. Several contextual factors, such as proximity to green spaces, the perceived safety of activity areas, and the availability of social support during PA, were also documented. Their influence on outdoor exercise participation among older adults is widely recognized. These variables were considered potential confounders or moderators, following the guidelines outlined in Strengthening the Reporting of Observational Studies in Epidemiology (STROBE) (Braillon & Naudet, 2023; Skrivankova et al., 2021).

Participants completed all questionnaires in Mandarin Chinese, using either self-administered or assistant-assisted formats, depending on their literacy level, visual acuity, and preference. The data collectors received standardized training on how to administer surveys and communicate effectively with older adults. The instruments were piloted with 20 participants to confirm their clarity and feasibility prior to implementation. Minor revisions were made to ensure accessibility and cultural appropriateness of the instruments.

### 2.4 Statistical analysis

At the initial stage of data cleansing, completeness was verified, logical coherence was ensured among questionnaire responses, and outliers were identified. All demographic and primary study variables were analyzed using descriptive statistics. Statistically, categorical data (such as gender and educational level) have been summarized as percentages, while ordinal and scale-based data have been characterized by means and standard deviations (mean ± SD), respectively. Significant differences between demographic characteristics were observed using an independent *t*-test or χ^2^ test (∗∗∗p < 0.001). For exploring relationships among key variables, point-biserial correlation coefficients were applied to assess associations between dichotomous variables (Rusakov, 2023; Shieh, 2021; Wang & Zhang, 2023) (e.g., morning vs. nighttime PA; green space vs. non-green space). In accordance with statistical standards for mixed data types, point-biserial correlations were used to investigate relationships between binary and continuous and ordinal variables (e.g., PA duration vs. depression disorder score).

The effects of PA timing (morning vs. nighttime), location (riverbank green space vs. non-green spaces), and their interaction on physical and mental health outcomes were assessed using generalized linear models (GLMs) (Choi et al., 2024; Gabriel et al., 2024; Maronge et al., 2023). Gamma distribution models with log links were applied to positively skewed variables (e.g., GAD-7 and PHQ-9 scores) in accordance with established methodologies. The models were adjusted for factors including age, gender, education, living conditions, employment conditions, and body function pain. Whenever substantial main effects were identified in GLMs, post-hoc comparisons with mean differences (MDs) and 95 % confidence intervals were conducted. When applicable, Cohen's d was applied to evaluate small, medium, and large effects (d = 0.2, 0.5, 0.8). Sensitivity analyses (Do et al., 2022) were performed to assess robust results by stratifying individuals based on PA timing (morning vs. nighttime) and location (riverbank green space vs. non-green spaces). Various regression models were constructed for each category to determine whether time, location, or both affected mental health outcomes. To ensure statistical stability, variance inflation factors (VIFs) were used to identify multicollinearity among predictors. Two-tailed p-values less than 0.05 were considered statistically significant. Multiple comparisons were mitigated with a Bonferroni correction. STROBE declaration criteria for observational studies were followed in all analyses. The statistical analyses were conducted using IBM SPSS Statistics 27.0 (IBM Corp., Armonk, NY, USA).

## 3 Results

### 3.1 Demographic and psychological attributes of participants

**Fig. 2:**
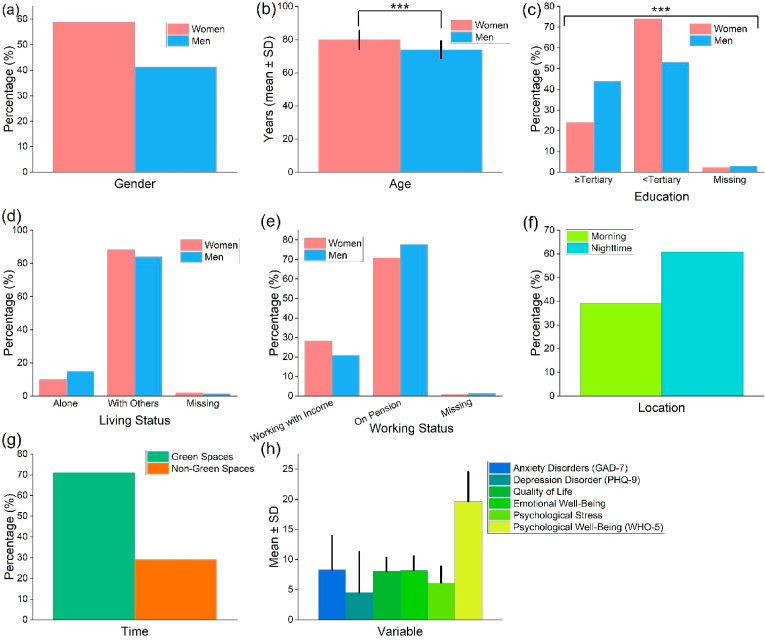
Panels (a–e) display demographic characteristics; panels (f–g) detail the location and timing of square dance activities; and panel (h) presents the psychological attributes of participants, all derived from urban riverbank green spaces in Mianyang, China. *Note*: Significant differences observed using an independent *t*-test or χ^2^ test (∗∗∗p < 0.001).

Fig. 2 presents a comprehensive overview of participant demographics, square dance preferences, and psychological health indicators. The sample consisted of 58.77 % women and 41.23 % men. Compared with men (73.9 ± 5.42), women had a significantly higher (p < 0.001) average age (79.83 ± 5.71). Women and men exhibited marked differences in their educational attainment (p < 0.001): 73.88 % of women did not pursue education beyond the tertiary level, compared to 52.99 % of men. Most participants lived with others, with cohabitation reported by 88.06 % of women and a slightly lower proportion of men (83.96 %). As far as occupational status is concerned, retirees constituted the largest subgroup, accounting for more than 70 % of both genders. In terms of preferred timing for square dancing, nearly 60 % of participants preferred nighttime sessions over morning ones (39.18 %). Additionally, a significant majority (71.08 %) expressed a preference for square dancing in green environments rather than non-green environments.

Among the measured metrics, psychological well-being (WHO-5) showed the highest scores (19.61 ± 4.86). Comparatively, anxiety (GAD-7) and depression (PHQ-9) disorders had lower scores (8.30 ± 5.6 and 4.51 ± 6.77, respectively). The measures of quality of life (8.06 ± 2.27), emotional well-being (8.19 ± 2.23), and psychological stress (6.11 ± 2.76) were all within moderate ranges.

### 3.2 Square dancing and participant psychology correlation analysis

**Fig. 3:**
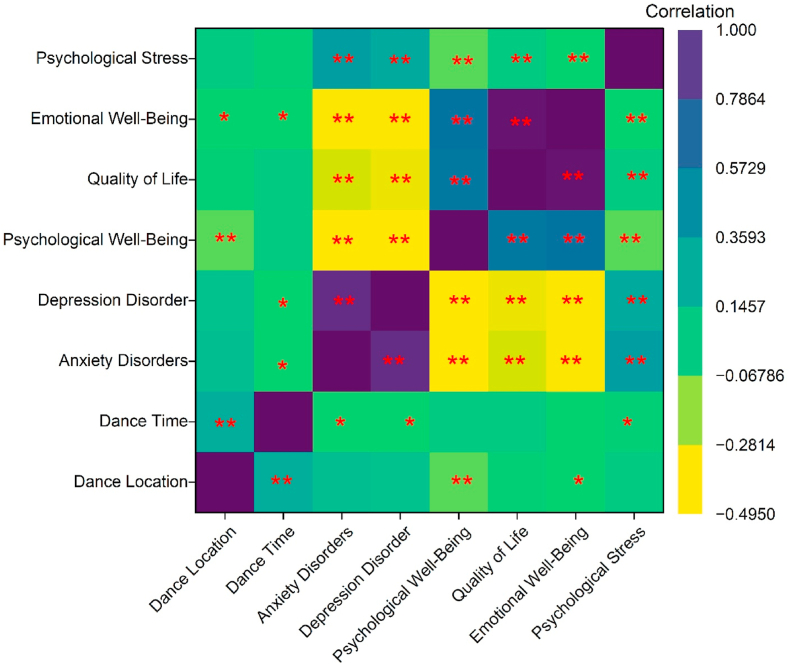
The point-biserial correlation matrix heatmap presents the relationships between dance-related variables and psychosocial health outcomes. Statistically significant associations are indicated (∗p < 0.05; ∗∗p < 0.01). *Note:* The activity of the square dance location was categorized as “green space = 0” (reference group) and “non-green space = 1”; the square dance activity time was categorized as “nighttime = 0” (reference group) and “morning = 1” for point-biserial correlation.

Fig. 3 presents a point-biserial correlation (r_pb_) heatmap that illustrates the relationships between dance-related PA and psychosocial health outcomes. Psychological characteristics and dance participation showed significant correlations (∗p < 0.05; ∗∗p < 0.01). Dance participation was associated with health disorder symptoms reduction. Furthermore, there was a positive correlation between dance participation in urban riverbank green venues and psychological and emotional well-being. The evidence suggests that dance activities could potentially improve PA, a factor with a negative correlation with depression and anxiety disorders.

This highlights that changing the environment from non-green to green spaces in which individuals dance can affect their mental health, emphasizing the importance of supportive or enjoyable dance settings (r = 0.155). Psychological (r = −0.206∗∗) and emotional well-being (r = −0.132∗∗) and psychological stress were found to be directly related to quality of life, suggesting that higher stress levels may be associated with poorer mental health. Both depression and anxiety disorders demonstrated substantial negative correlations with quality of life (r = −0.407∗∗ and r = −0.376∗∗) and emotional well-being (r = −0.448∗∗ and r = −0.432∗∗), respectively, indicating that individuals with mood and anxiety disorders experience adverse psychological effects. Psychological (r = 0.530∗∗) and emotional well-being (r = 0.934∗∗) were strongly correlated with quality of life, suggesting that those with greater well-being perceive their lives more positively. PA, through dancing, appears to benefit mental wellness and quality of life.

### 3.3 The influence of square-dancing time and space on psychological characteristics

**Fig. 4:**
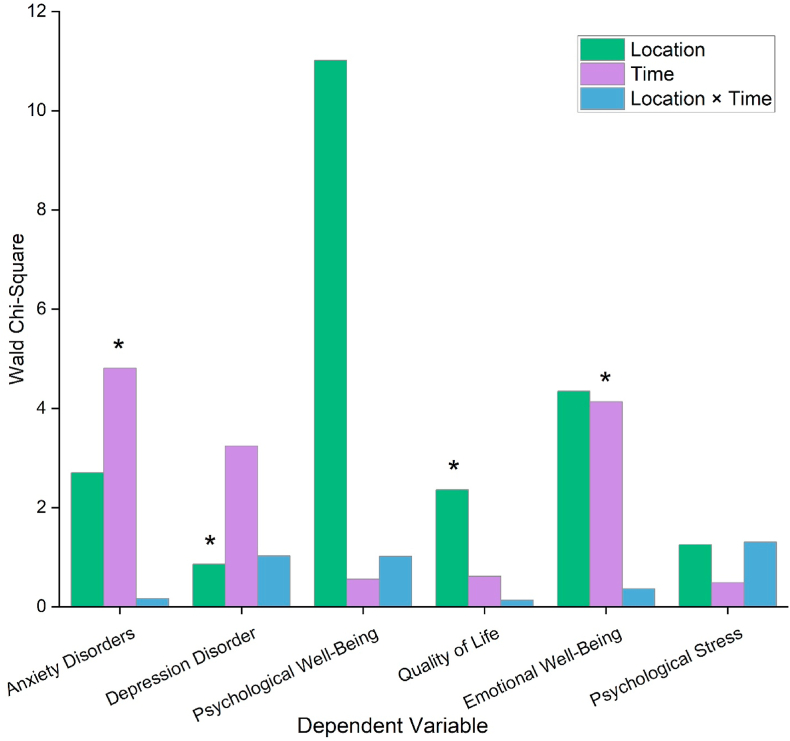
Bar graphs presenting the results of Wald Chi-Square tests examining the effects of square dance location, timing, and their interaction on psychological health indicators. Asterisks (∗p < 0.05) denote statistically significant outcomes. *Note:* Every analysis was adjusted for demographic factors.

Fig. 4 shows the results of Wald Chi-Square tests on various psychological outcomes based on PA location, time, and interaction. Each dependent variable (e.g., anxiety disorders, psychological well-being) was analyzed across three parameters, separated by different colors: green for location, purple for time, and blue for location × time interaction. Asterisk bars indicate statistically significant effects (∗p < 0.05). PA time was associated with higher Wald Chi-Square values for anxiety (Wald χ^2^ = 4.811∗) and depression disorders (Wald χ^2^ = 3.242). PA time significantly predicted reduced symptoms in both areas. Conversely, PA venue location (green space) presented the highest Wald Chi-Square value (Wald χ^2^ = 11.014∗), which reflects a strong correlation between improvements in psychological well-being and dance green space venue. The chi-square values indicated that location also significantly influences quality of life (Wald χ^2^ = 2.360) and emotional well-being (Wald χ^2^ = 4.131∗). The environment in which PA occurs has a critical impact on emotional well-being (Wald χ^2^ = 4.348 for location and Wald χ^2^ = 4.131∗ for time). Across all outcomes, location and time interaction (blue bars) generally showed minimal effects. Time and place both impact psychological health, but their combined effect or interaction has not been statistically significant. Dance activities alleviate psychological concerns and improve well-being, regardless of their time and location.

### 3.4 Sensitivity analysis of square-dancing time and space

**Fig. 5:**
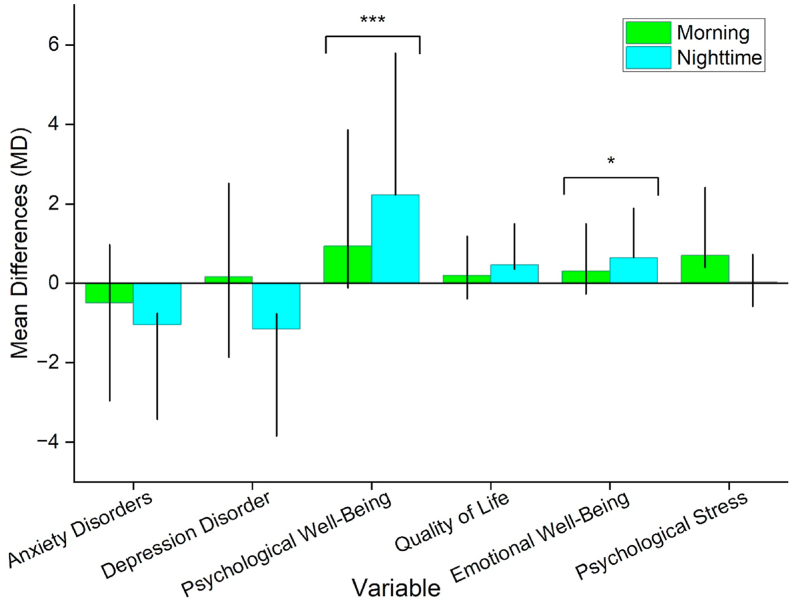
Bar graphs of mean differences and corresponding confidence intervals (depicted as vertical bars) for psychological health outcomes among older adults engaging in morning versus nighttime square dancing within urban green spaces *with reference to non-green spaces*. Asterisks (∗∗∗p < 0.001 and ∗ p < 0.05) denote statistically significant outcomes. *Note:* Every analysis was adjusted for demographic factors.

Fig. 5 shows the MD in six psychological variables depending on when participants perform PA—morning (green bars) versus nighttime (cyan bars). The graph indicates the direction and magnitude of impacts on psychological outcomes, with error bars representing variability and asterisks denoting statistical significance (∗p < 0.05, ∗∗∗p < 0.001). Psychological well-being has been observed to be the most significant benefit (MD = 0.937∗∗∗ [95 % CI = −1.049–2.922] ∼ 2.227∗∗∗ [95 % CI = 0.891–3.564]). Participants who performed the PA at nighttime showed a significantly greater positive mean difference than those who performed the dance in the morning. The difference has statistical significance, indicating that nighttime PA was associated with improved psychological well-being (MD = 0.937∗∗∗). Similarly, nighttime square dance consistently reported higher emotional well-being scores (MD = 0.641∗ [95 % CI = 0.0286–1.254]) than their morning counterparts (MD = 0.308∗ [95 % CI = −0.575–1.191]), though the trend is less pronounced. Despite its significance, this difference shows that nighttime PA may improve emotional well-being. Both morning (MD = 0.198 [95 % CI = −0.588–0.984]) and nighttime (MD = 0.463 [95 % CI = −0.108 to 0.035]) groups demonstrated improvements in quality of life, with the nighttime group showing the greatest benefit. However, the error bars suggest that the apparent disparity was not statistically significant. While anxiety disorders, depressive disorders, and psychological stress did not differ significantly between morning and nighttime PA, trends indicate somewhat improved outcomes for nighttime participants, particularly with respect to anxiety and depression disorders. Psychological effects may be influenced by square dance time. Specifically, nighttime PA appears to improve psychological and emotional well-being, illustrating the importance of timing in therapeutic or recreational dance interventions.

**Fig. 6:**
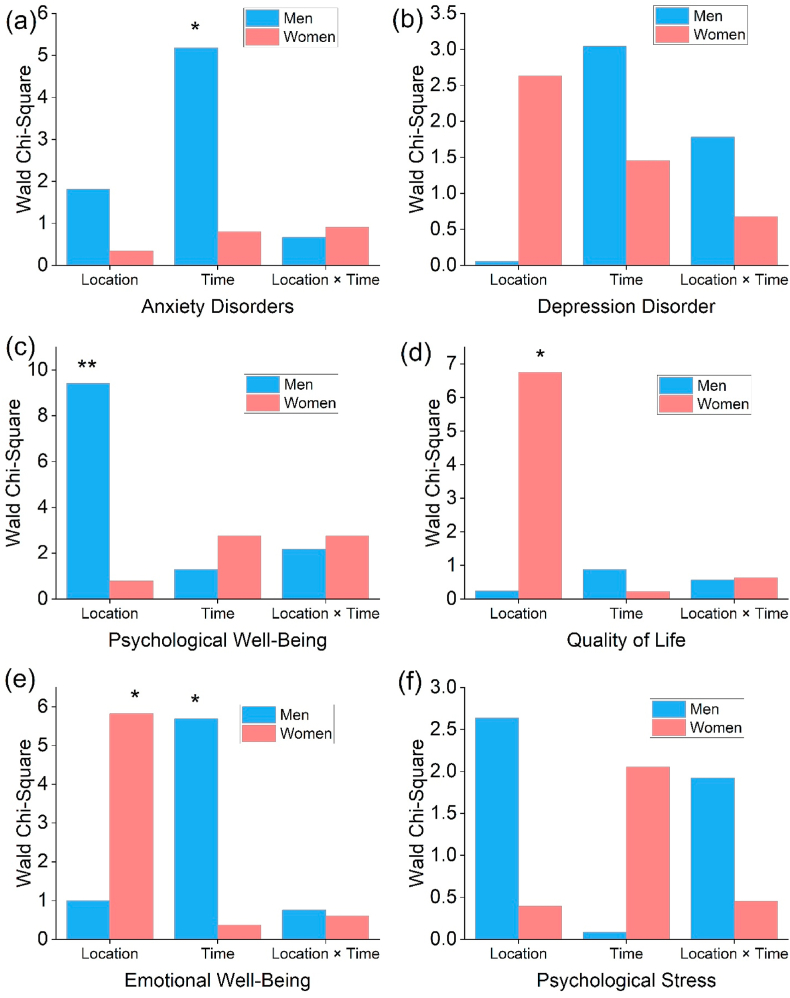
Bar graphs (a–f) provide gender-specific Wald Chi-Square analyses of the effects of square dance location, timing, and their interaction on various psychological health outcomes. Asterisks (∗∗p < 0.01 and ∗ p < 0.05) denote statistically significant outcomes. *Note:* Every analysis was adjusted for demographic factors.

Fig. 6 compares six psychological outcomes based on square dance location, timing, and interaction. Blue bars represent men, and red bars represent women, displaying Wald Chi-Square values for each component. Asterisks (∗∗p < 0.01 and ∗p < 0.05) indicated statistically significant effects.

Concerning anxiety disorders, square dance time influenced anxiety outcomes more profoundly in men (Wald χ^2^ = 5.179∗). Compared to men, women showed negligible effects across all three parameters. Across all three components of depression disorder, results revealed moderate chi-square values for both genders, but no statistically significant effects. Psychological well-being differed significantly by gender. Statistics indicated that psychological well-being in men was significantly influenced (Wald χ^2^ = 9.411∗∗) by their PA location (i.e., green spaces). In contrast, men showed decreased values across the other two variables. Location (i.e., green spaces) significantly affected the quality of life for women (Wald χ^2^ = 6.748∗), but not for men. Hence, the PA environment may significantly impact the quality of life reported by women. Both genders report the notable impact of location (Wald χ^2^ = 5.822∗ in women) and time (Wald χ^2^ = 5.689∗ in men) on emotional well-being. The data indicated that PA enhances emotional well-being regardless of gender, with women more affected than men. For females, location and interaction combinations have notably diminished effects on psychological stress. These areas do not differ statistically.

## 4 Discussion

Researchers examined the impacts of participating in PA, particularly square dancing, at different times of the day (morning versus night) and in different locations (green spaces versus non-green spaces) on the mental and physical well-being of older adults in urban riverside areas of China. Statistical analysis revealed that nighttime PA in urban green spaces was significantly correlated with improved psychological well-being, enhanced emotional health, and an overall higher quality of life (Fig. 6). PA timing was more strongly associated with anxiety levels among men participants. In contrast, PA location was more strongly associated with emotional health and quality of life outcomes among women participants. These findings support the positive effects of nighttime PA in green environments, as well as the significant differences between men and women. Gender differences may stem from sociocultural norms. Men prefer morning routines that emphasize discipline, while women respond more to green spaces for their aesthetics and perceived nighttime safety. Traditional roles and unequal public space access may explain why location impacts women more, and timing affects men more.

This study provides novel insights by incorporating square dancing into the literature regarding the health benefits of physical exercise and green spaces. We placed special emphasis on timing and location. Previous studies consistently demonstrated the numerous positive effects of regular exercise on both physical and mental health (Jiang et al., 2025; Menotti & Puddu, 2025; VaezMousavi et al., 2025). These benefits include reduced risks of chronic illness, enhanced cognitive abilities, relief of depression and anxiety symptoms, and improved psychological well-being (Kong et al., 2025; Serra & Feio, 2024). However, our focus was on comparing morning and nighttime physical activities in green urban environments in the context of square dancing. This is a topic that the literature has not thoroughly examined. This unique perspective adds substantial depth to our understanding of how timing and location affect PA effectiveness in these settings.

It is particularly noteworthy that our research identified a significant positive correlation between nighttime PA and psychosocial outcomes, such as psychological well-being and emotional health (Fig. 6). One possible explanation for these benefits is that the atmosphere of green spaces at night is quieter and less distracting. The benefits of such an environment could be greater than those of normal day-to-day settings in terms of mental recovery and stress reduction (Neil-Sztramko et al., 2022). In addition, nighttime activities in urban environments often provide opportunities for social support and engagement, contributing to their positive psychological and emotional effects (Cook et al., 2025; Yuan & Chen, 2025; Zhang et al., 2024). The tradition of square dancing among older adults in China promotes social relationships, making it an especially beneficial activity for psychological well-being.

The significant correlation observed between the placement of square dancing (green spaces versus non-green spaces) and the improvement of psychological well-being is in agreement with previous research that highlights the healing potential of natural settings (Cao et al., 2021; Li et al., 2022; Wu et al., 2024). The visual appeal of natural green spaces, like urban riverbanks, as well as the opportunity for social interaction, may encourage physical exercise (Petersen et al., 2018). This type of activity enhances psychological health. Gender-specific findings have added to our understanding, providing valuable insights. Prior to this, Simandan (2021) argued that gender in population health is dynamic, shaped by social roles, mobility limitations, and safety perceptions. Earlier, Simandan (2011) explored how time, place, and well-being influence health behaviors through a temporal-spatial lens. Observations revealed that male anxiety was more sensitive to PA timing. This finding suggests that gender differences may exist in routines, perceptions of safety, and expectations of social behavior. Conversely, the location of the PA had a significant impact on the quality of life and emotional well-being of women. It may be that their perception of safety and emotional attachment to green spaces differs. Results of previous studies have indicated that women perceive natural landscapes as safe and psychologically soothing environments (Jiang et al., 2025), which can enhance their health. Post-COVID-19 data from 2023 showed older Chinese adults reengaged in communal activities like square dancing to restore their physical and mental health. Prolonged restrictions lead to sedentary habits and distress (Quinn et al., 2025; Simandan, Rinner, & Capurri, 2024), highlighting square dancing as a key recovery tool.

### 4.1 Policy implications

Our study has significant implications for both public health and community planning. Considering the many benefits of nighttime PA in green spaces, urban planning efforts may emphasize the creation of accessible, safe, and inviting environments that encourage evening participation, particularly among senior citizens. It is possible to enhance lighting, improve security measures, and provide facilities for evening communal activities, such as square dancing, as part of such initiatives. In terms of public health, promoting regular nighttime PA in natural settings for elderly adults could be an effective strategy for improving psychological health. To promote community involvement, psychological well-being, and social connection, it is worthwhile to incorporate culturally relevant group activities into health promotion initiatives, particularly square dancing. Furthermore, gender-specific findings indicate that targeted strategies are necessary to encourage PA while considering the individual constraints and incentives men and women face regarding timing and location.

### 4.2 Study limitations

Analyzing the present research results involves several constraints. We were unable to establish causal relationships between PA timing and location and observed health outcomes due to the cross-sectional research design. Although we found significant connections, longitudinal studies or intervention studies are necessary to confirm the directionality of these interactions. Furthermore, the self-report instruments used to measure PA and psychosocial outcomes may have introduced biases related to recall and social desire. In future research, objective measures such as accelerometers for PA and clinical assessments for psychological issues may be used to enhance the validity of the findings. Finally, the generalizability of these results may be limited by their specific cultural and environmental context, as well as their focus on square dancing activities. While these findings are culturally relevant, they may not be easily transferable to other contexts or forms of PA.

### 4.3 Future research directions

Future research may address the limitations outlined above and investigate the mechanisms underlying the relationship between nighttime participation in green spaces and psychological health outcomes. Researchers are encouraged to use longitudinal and experimental designs to establish causality and identify mediating variables, such as perceived safety, social support, and environmental quality. Additionally, studies may include a diverse range of urban and rural populations to extend the demographic scope. Furthermore, they may examine other forms of PA besides square dancing to enhance their generalizability. To further strengthen public health initiatives, it would be beneficial to study the long-term adherence and enduring psychological effects of nighttime PA interventions.

## 5 Conclusion

This study examined the impact of timing (morning versus nighttime) and location on the psychosocial benefits of square dancing for older adults living near urban riverbanks in China. Participants in the study indicated that nighttime PA, particularly square dancing in green areas, contributed significantly to enhanced psychological well-being, emotional health, and overall quality of life. It appears that nighttime PA within natural urban environments may serve as an effective and culturally appropriate intervention for improving the psychosocial benefits of older Chinese adults. Gender-specific analysis showed PA timing and location had distinct effects. Among men, anxiety levels were significantly correlated with PA timing. For women, quality of life and emotional well-being were strongly associated with PA locations. These differences show that PA programs for older adults require gender-sensitive design and implementation. Considering community planning and public health perspectives, the results supported the creation of safe, accessible, and attractive green spaces that encouraged group activities at night. These insights are encouraged for healthcare professionals and policymakers to utilize them to promote culturally relevant, community-centered physical activities for older adults, such as evening square dancing, potentially increasing participation rates and improving psychological well-being. To clarify causal relationships and the processes behind them, further longitudinal or experimental studies are required. Research of this nature is essential to developing targeted strategies to address health challenges faced by aging populations in rapidly urbanizing regions such as China.

## CRediT authorship contribution statement

**Song Wei:** Writing – review & editing, Writing – original draft, Visualization, Validation, Supervision, Software, Resources, Project administration, Methodology, Investigation, Funding acquisition, Formal analysis, Data curation, Conceptualization. **Hongli Yu:** Writing – review & editing, Writing – original draft, Visualization, Validation, Supervision, Software, Resources, Project administration, Methodology, Investigation, Funding acquisition, Formal analysis, Data curation, Conceptualization. **Chen Sun:** Writing – review & editing, Writing – original draft, Visualization, Validation, Supervision, Software, Resources, Project administration, Methodology, Investigation, Funding acquisition, Formal analysis, Data curation, Conceptualization.

## Ethical statement

The study was conducted in accordance with the Declaration of Helsinki and approved by the Institutional Review Board (or Ethics Committee) of Yibin University, China (November 17, 2022).

## Declaration of generative AI and AI-assisted technologies in the writing process

During the preparation of this work, the author(s) used Wordtune and Grammarly in order to fix grammatical errors and enhance understanding of context and meaning. After using this tool or service, the author(s) reviewed and edited the content as needed and took full responsibility for the content of the publication.

## Funding

This research was funded by the College of Physical Education and Health Science, 10.13039/501100008006Yibin University, Yibin 644000, Sichuan, China (No. 2021YY11); the 10.13039/501100004858Sichuan University of Science and Engineering (No. 2024RC093); and the Zigong City Philosophy and Social Science Key Research Base National Physical Fitness and Sports Industry Research Center (NO. GT-02202417).

## Declaration of competing interests

The authors declare that they have no known competing financial interests or personal relationships that could have appeared to influence the work reported in this paper.

## Data Availability

Data will be made available on request.
